# Flexible Piezoresistive Sensor Based on CNT/PVA Composite with Wide Linear Detection Range for Human Motion Monitoring

**DOI:** 10.3390/polym17101378

**Published:** 2025-05-17

**Authors:** Lijun Chen, Yucheng Huang, Honglong Ning, Yuxiang Liu, Huacheng Tang, Rui Zhou, Shaojie Jin, Jiahao Zheng, Rihui Yao, Junbiao Peng

**Affiliations:** 1 Guangdong Basic Research Center of Excellence for Energy & Information Polymer Materials, State Key Laboratory of Luminescent Materials and Devices, School of Materials Sciences and Engineering, South China University of Technology, Guangzhou 510640, China; 202230270202@mail.scut.edu.cn (L.C.); yucheng_h@163.com (Y.H.); lyx19924688627@163.com (Y.L.); 202421021262@mail.scut.edu.cn (R.Z.); 18770602788@163.com (S.J.); 202321020959@mail.scut.edu.cn (J.Z.); psjbpeng@scut.edu.cn (J.P.); 2 The International School of Microelectronics, Dongguan University of Technology, Dongguan 523808, China; 3 Key Laboratory of Guangdong Province for High Property and Functional Polymer Materials, South China University of Technology, Guangzhou 510640, China

**Keywords:** flexible piezoresistive sensor, CNT/PVA composite, microstructures, human motion monitoring and analysis

## Abstract

In recent years, flexible pressure sensors have attracted significant attention due to their extensive application prospects in wearable devices, healthcare monitoring, and other fields. Herein, we propose a flexible piezoresistive sensor with a broad detection range, utilizing a CNT/PVA composite as the pressure-sensitive layer. The effect of the CNT-to-PVA ratio on sensing performance was systematically investigated, revealing that the sensor’s sensitivity initially increases and then decreases with rising CNT content. When the weight percentage of CNTs reaches 11.24 wt%, the sensing film exhibits optimal piezoresistive properties. A resistance model of the composite conductive material was established to elucidate the sensing mechanism associated with CNT content in detail. Furthermore, hill-like microstructures were fabricated on a PDMS substrate using sandpaper as a template to further enhance overall performance. The sensor demonstrates a sensitivity of 0.1377 kPa^−1^ (<90 kPa), a sensing range of up to 400 kPa, a response time of 160 ms, and maintains excellent stability after 2000 folding cycles. It can accurately detect human joint flexion and muscle activity. This work is expected to provide a feasible solution for flexible electronic devices applied in human motion monitoring and analysis, particularly offering competitive advantages in applications involving wide-range pressure detection.

## 1. Introduction

Flexible pressure sensors have demonstrated significant application potential in health monitoring, electronic skin, human–machine interaction, and other various fields, making them a prominent research focus [1,2,3,4,5,6]. Based on their working mechanisms, flexible pressure sensors can be categorized into piezoresistive, capacitive, piezoelectric, and triboelectric types [7,8,9]. Piezoresistive and capacitive sensors require external power sources to work, whereas piezoelectric and triboelectric sensors can operate as self-powered devices without external power. Among these, flexible piezoresistive sensors have attracted extensive attention due to their simple structure, ease of fabrication, and excellent performance [10,11]. Current research primarily focuses on improving sensitivity but often sacrifices the detection upper limit (<10 kPa) [11,12]. However, most daily human activities, such as joint loading and exercise impact forces, involve pressures far exceeding 10 kPa [13,14,15]. Solely pursuing high sensitivity while neglecting the detection range hinders the application of sensors in diverse complex scenarios [16,17]. Consequently, there is an urgent need for sensing solutions that combine a wide detection range with high reliability in fields such as medical diagnostics, motion analysis, and injury rehabilitation. In this study, we strive to propose a high-reliability, flexible pressure sensor with a wide sensing range that enables various applications in scenarios such as human motion monitoring.

The selection of active materials is crucial in determining the performance of flexible sensors. Typically, active materials used for fabricating sensing layers mainly include metallic materials (e.g., AgNWs), carbon-based materials (e.g., graphene, carbon nanotubes), and intrinsically conductive polymers (e.g., PPy) [7,8,9,10,11]. Among these, carbon-based materials have become the most favored choice owing to their exceptional electronic and mechanical properties, excellent processability, and outstanding pressure sensitivity [14,18,19]. A common strategy involves directly fabricating carbon-based materials into films to achieve sensing functionality. Wang et al. [20] uniformly sprayed CNT solution onto paper to create paper-based CNT films, which were then sandwiched between polydimethylsiloxane (PDMS) films and polyimide (PI) films with interdigital electrodes to fabricate sensors. Liu et al. [1] prepared graphene films on microstructured PDMS substrates through a spin-coating method, developing a high-performance flexible sensor. The fabricated sensor simultaneously exhibited a high sensitivity (4.56 kPa^−1^, 5–20 kPa), a wide detection range (~250 kPa), and a fast response time (130 ms). In addition, numerous recent studies have reported the application of carbon-based materials for damage sensing and structural health monitoring [21,22,23]. The underlying sensing mechanisms primarily involve fracture behavior and crack propagation under external mechanical loading, accompanied by a corresponding change in contact resistance and tunneling effect. These practical applications demonstrate the exceptional sensing capabilities and diverse physical transduction mechanisms of carbon-based materials. However, carbon-based films prepared by conventional solution methods often exhibit inherent brittleness and susceptibility to fracture. Under high pressure or bending, these films are prone to cracking due to stress concentration, which compromises sensor stability and reliability and limits their applications across various fields [24,25,26,27].

To address this issue, researchers have attempted to develop composite conductive materials with enhanced mechanical properties. Incorporating carbon-based conductive fillers into polymer matrices to prepare composite materials has proven to be an effective approach [28,29,30]. This method serves two key purposes: (1) it establishes a conductive network within the polymer matrix [24], preserving the material’s electrical conductivity and enabling electrical response to pressure; and (2) it significantly improves the material’s mechanical properties [31], endowing it with excellent bending and tensile strength to withstand broader pressure ranges and better adapt to practical applications of flexible sensors. Zhao et al. [32] prepared CNT/PDMS composite film with a hierarchical structured surface (h-CNT/PDMS) through solution blending and blade-coating methods, which were then assembled with interdigital electrodes and polyurethane (PU) tape to fabricate flexible pressure sensors. Xiao et al. [33] employed graphene nanoplatelets (GNPs)/PDMS as the composite conductive material and utilized direct ink writing (DIW) technology to directly mold sensitive units onto PDMS substrates, creating flexible piezoresistive sensors. Xu et al. [34] developed porous PDMS-carbon black (CB) composites via a solvothermal method using spherulite NaCl (s) templates as pore-forming agents. The resulting flexible piezoresistive sensor demonstrated high sensitivity (0.52 kPa^−1^) across a pressure range of 0.026–100 kPa, along with cycling stability exceeding 7500 cycles. However, commonly used polymer matrices such as PDMS often exhibit creep and mechanical energy dissipation due to their high viscoelasticity and weak interfacial interactions. While these materials improve device mechanical stability, they limit sensitivity and demonstrate response hysteresis [8,28,35].

Compared with other low-dimensional carbon materials, CNTs possess unique advantages as conductive fillers. Their higher aspect ratio facilitates the construction of conductive networks within polymer matrices, promoting charge transport and reducing the percolation threshold [24,31]. However, CNTs tend to agglomerate due to strong van der Waals forces, which makes the uniform dispersion of CNTs in polymers a critical factor determining the composite’s performance [36,37]. Polyvinyl alcohol (PVA), a polymer with excellent film-forming capability and stability, demonstrates superior water solubility that enables the facile preparation of CNT/PVA composites through simple solution mixing. The resulting composites can be processed into pressure-sensitive films via wet coating techniques such as drop-coating or spin-coating. The hydroxyl (–OH) groups in PVA molecular chains can form hydrogen-bond networks with covalently modified CNTs (e.g., hydroxylated CNTs), which enhances interfacial interactions, effectively suppresses CNT agglomeration [38,39], and helps form stable composite materials. Furthermore, the weaker viscoelasticity of PVA potentially improves the pressure sensitivity and response speed of composite materials. Therefore, selecting PVA as the polymer matrix and embedding CNTs as conductive fillers, while rationally optimizing their ratio, may lead to the development of structurally stable and high-performance sensing materials.

In summary, we selected CNTs and PVA to prepare composite materials as sensing films, achieving a highly stable, flexible piezoresistive sensor with a wide linear working range. PDMS, possessing excellent flexibility, stretchability, and chemical stability, serves as the most widely used flexible substrate material. In this study, we prepared CNT/PVA functional layers on PDMS substrates through a drop-coating process and assembled them with interdigital electrodes to fabricate flexible sensor devices. The piezoresistive characteristics of sensors with different CNT-to-PVA ratios were systematically compared and analyzed, accompanied by a comprehensive explanation of the working mechanism. By employing sandpaper as a template, we created flexible substrates with hill-like microstructures, further enhancing the sensor’s sensitivity and detection range. The optimized sensor demonstrates a sensitivity of 0.1377 kPa^−1^ within 90 kPa and 0.0353 kPa^−1^ up to 400 kPa, along with excellent repeatability over 1200 loading/unloading cycles. Benefiting from the outstanding mechanical strength of the CNT/PVA composite and its stable conductive network, the sensor maintains consistent loading/unloading stability even after 2000 folding cycles, exhibiting remarkable reliability and durability. The sensor successfully detects various human joint movements (finger and knee flexion) and muscle activities (arm and leg muscle contractions). This work presents a sensor with simple fabrication processes, exceptional performance, and cost-effective environmental friendliness, showing significant application potential in wearable devices and motion health monitoring fields.

## 2. Materials and Methods

### 2.1. Raw Materials

PDMS base and Sylgard 184 curing agent were provided by Dow Corning Co., Ltd. (Midland, MI, USA); hydroxylated multi-walled carbon nanotubes (MWCNTs) were supplied by Shenzhen Suiheng Technology Co., Ltd. (Shenzhen, China); polyvinyl alcohol (PVA) was obtained from Wuxi Yatai United Chemical Co., Ltd. (Wuxi, China).

### 2.2. Preparation of Flexible PDMS Substrates

**Figure 1:**
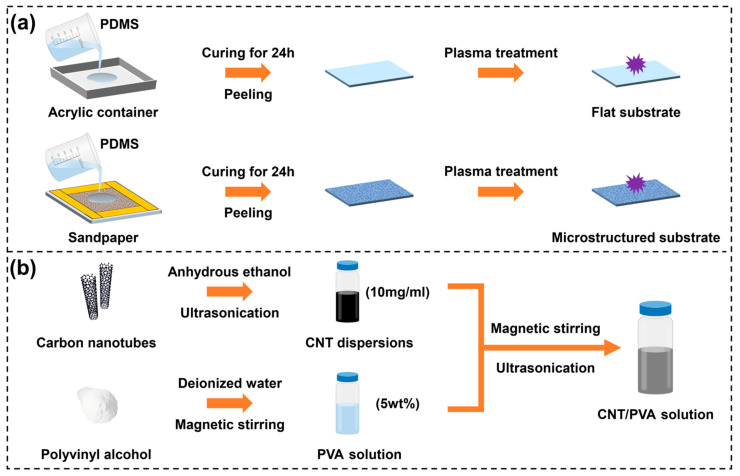
Fabrication process flow: (**a**) flat substrate and microstructured substrate; (**b**) solution preparation.

As shown in Figure 1a, PDMS base and Sylgard 184 curing agent were mixed at a 10:1 ratio, then degassed by ultrasonic stirring for 20 min. The PDMS solution was poured into clean acrylic containers and cured at room temperature for 24 h to obtain flat substrates (FS). For microstructured substrates (MS), 800-grit sandpaper was cut to an appropriate size and fixed on clean glass with tape. PDMS solution was poured onto the sandpaper surface and cured at room temperature for 24 h. All PDMS substrates were cut into 1 × 1 cm^2^ pieces, cleaned with deionized water and anhydrous ethanol to remove surface contaminants, air-dried, and treated with oxygen plasma for 2 min.

### 2.3. Preparation of CNT Dispersions, PVA Solution, and CNT/PVA Solution

As shown in Figure 1b, 0.2 g of hydroxylated CNTs was added to 20 mL of anhydrous ethanol, followed by ultrasonic dispersion at 100 W power and 35 kHz operating frequency for 30 min at room temperature to obtain a 10 mg/mL CNT dispersion. Subsequently, 5 g of white PVA powder was added to 95 mL of deionized water and magnetically stirred at 90 °C for 2 h to ensure complete dissolution of the PVA powder. The dissolved PVA solution was then left to cool at room temperature to eliminate bubbles, resulting in a 5 wt% PVA aqueous solution. The 10 mg/mL CNT ethanol dispersions and 5 wt% PVA aqueous solution were mixed at volume ratios of 2:1, 3:2, 1:1, 2:3, and 1:2, respectively. Each mixture was magnetically stirred for 30 min at room temperature, followed by an additional 30 min of ultrasonic treatment to obtain uniformly dispersed CNT/PVA solutions.

### 2.4. Fabrication of Interdigital Electrodes

The designed interdigital electrodes featured 3 finger pairs with 0.8 mm finger width and 0.7 mm spacing. PET tape was cut to size, and the protective film was removed to serve as the electrode substrate. Conductive silver paste was screen-printed through a 200-mesh stencil to form electrode patterns on PET film. Printed electrodes were dried at 90 °C for 45 min in a drying oven to evaporate solvents, yielding firmly adhered interdigital electrodes.

### 2.5. Fabrication of Sensing Films and Sensors

**Figure 2:**
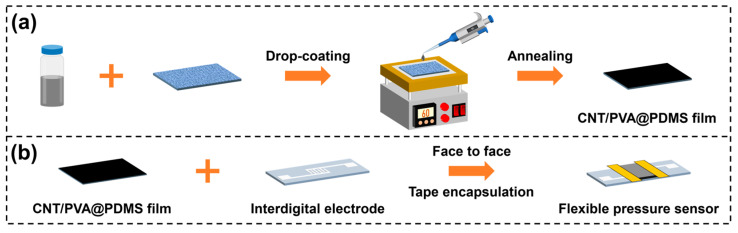
Fabrication process flow: (**a**) CNT/PVA sensing film; (**b**) flexible pressure sensor.

The CNT/PVA sensing films were fabricated using a drop-coating and drying method. For each sample, 30 μL of CNT/PVA solution (at various ratios) was dropped onto oxygen plasma-treated PDMS substrates, followed by annealing at 60 °C for 15 min on a hotplate to form CNT/PVA films (Figure 2a). Subsequently, the films were precisely aligned face-to-face with interdigital electrodes and secured using tape to complete the sensor assembly (Figure 2b). The fabricated sensor samples were systematically labeled as shown in Table 1.

### 2.6. Characterization and Testing Equipment

The surface morphology of the films was characterized using a laser confocal microscope (OLS5000-SAF, OLYMPUS, Hamburg, Germany). The sheet resistance of CNT/PVA films was measured by a four-point probes measurement system (RTS-9, Guangzhou Four-Point Probes Technology, Guangzhou, China). Film thickness was determined using a step profiler (Dektak 150, Veeco, Plainview, NY, USA). Sensor performance was evaluated using a digital multimeter (DMM-6500, Keithley, Cleveland, OH, USA) and a universal testing machine (ZQ990A, Dongguan Zhituo Precision Instrument, Dongguan, China).

## 3. Results and Discussion

### 3.1. Characterization of CNT/PVA@PDMS Films

**Figure 3:**
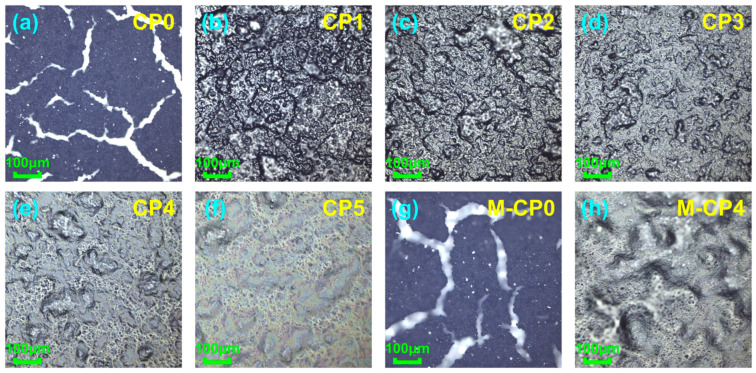
Laser confocal microscopy images at 20× magnification: (**a**–**f**) sensing films of CP0–CP5; (**g**,**h**) sensing film of M-CP0 and M-CP4.

The prepared films were characterized using laser confocal microscopy. Figure 3a and Figure 3g, respectively, display the surface morphology of CNT films fabricated on flat and microstructured substrates through identical drop-coating and drying processes. Figure 3b–f,h present the surface micro-morphology of sensing films of CP1–CP5 and M-CP4. The results reveal that pure CNTs cannot form continuous films but instead fracture into numerous fragments. This phenomenon primarily occurs because the high concentration of pure CNT dispersions leads to significant agglomeration, where CNT clusters connect only through van der Waals forces or physical entanglement, creating numerous defects within the film. Additionally, the rapid and non-uniform evaporation of ethanol solvent generates substantial film shrinkage stress, which is sufficient to rupture the CNT film and cause cracking. The incorporation of PVA enables the formation of intact films, attributable to two key factors: (1) PVA increases solution viscosity, slowing solvent drying rate and effectively reducing film shrinkage stress; (2) PVA acts as a binder, forming an interpenetrating and interconnected stable structure with CNTs. This structure not only enhances the binding force between CNTs but also facilitates stress buffering and distribution through load transfer within the materials. Furthermore, the introduction of PVA strengthens interfacial adhesion with the PDMS substrate, promoting the formation of continuous and robust films.

In Figure 3, the darker, gray-black areas represent regions with higher CNT concentration, while the lighter, yellowish-white areas indicate regions with higher PVA concentration. The alternating distribution of dark and light colored regions demonstrates the uniform dispersion of CNTs within the PVA matrix. It should be emphasized that this refers to the uniform distribution of numerous extremely small CNT clusters throughout the composite material. We can use coverage to characterize the extent to which the composite material is coated and filled by conductive materials, defined as the ratio of the area covered by conductive fillers to the total area [40]. As the CNT content increases, the film color progressively darkens, indicating an expansion of the surface area covered by CNTs. Generally speaking, higher coverage of conductive fillers leads to stronger electrical conductivity of the composite, but may result in reduced mechanical properties due to excessive aggregation of high-content fillers, causing stress concentration. The films prepared in this experiment exhibit similar characteristics: CP1 films show lower resistance values but are prone to tearing and damage under external forces, while CP5 films have much higher resistance but demonstrate greater toughness. Therefore, maintaining the concentration of conductive fillers within an appropriate range is essential to achieve a balance between the electrical conductivity and mechanical properties of the composite material.

**Figure 4:**
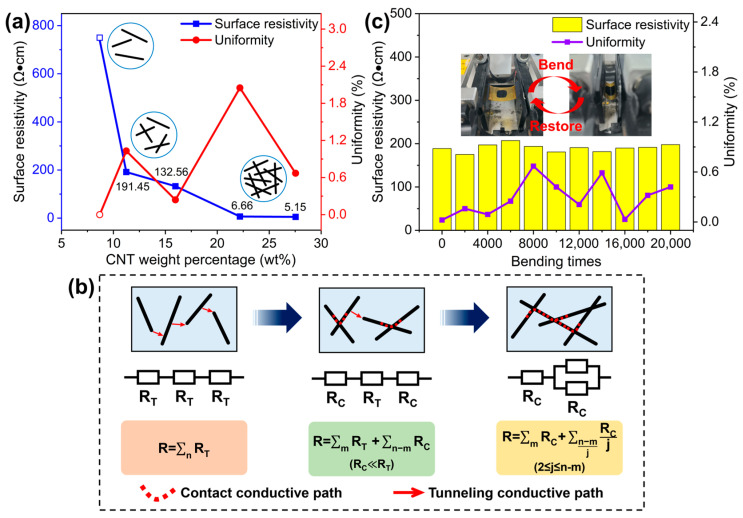
(**a**) Surface resistivity of CNT/PVA films; (**b**) resistance model of conductive paths evolution in CNT/PVA composite with increasing CNT weight percentage; (**c**) variation of surface resistivity in CNT/PVA composite during 20,000 bending cycles.

The thickness of CNT/PVA films for each sensor was measured by a step profiler, showing that the thickness was basically around 30 μm. The sheet resistance of CP1–CP5 films was measured by the four-point probes method, and the surface resistivity ρ was calculated according to Formula (1):

(1) $$\rho ={\;R}_{s}\times \;\omega$$

where R_S_ is the sheet resistance and ω is the film thickness. Then, the volume ratio of the solution was converted into the weight percentage of CNTs in the composite material as the horizontal coordinate for plotting, as shown in Figure 4a. It can be observed that the surface resistivity of CNT/PVA films shows a significant nonlinear decrease with increasing CNT weight percentage. Figure 4b explains the reason for the nonlinearity through finite element analysis. A resistance model was constructed and analyzed by selecting a finite number *n* of carbon nanotubes in any microscopic unit area of the film. When the CNT weight percentage is less than 10 wt%, the CNTs as conductive fillers are almost separated from each other, and current conduction mainly occurs through the tunneling effect between a few CNTs that are relatively close to each other. Since there are very few positions where tunneling occurs, the total resistance can be approximately regarded as the series connection of *n* tunneling resistances R_T_ according to Formula (2):

(2) $$R=\sum_{n}R_{T}$$

Resulting in very high film resistivity. When the CNT weight percentage increases to the range of 10–12.5 wt%, some CNTs begin to come into contact to form a conductive network, and the total resistance can be regarded as the series connection of *m* tunneling resistances R_T_ and *n* − *m* contact resistances R_C_ according to Formula (3):

(3) $$R=\sum_{m}R_{T}+\sum_{n-m}R_{C}\;(R_{C}\;\ll {\;R}_{T})$$

Since R_C_ is much smaller than R_T_, the resistivity drops sharply in this zone, which is usually called the percolation zone [31,41,42]. As the CNT content continues to increase, the contact resistance R_C_ is connected through both series and parallel ways according to Formula (4):

(4) $$R=\sum_{m}R_{C}+\sum_{\frac{n-m}{j}}\frac{R_{C}}{j}(2\;\leq \;j\;\leq \;n-m)$$

where *j* represents that *n* − *m* R_C_ are connected in parallel in groups of *j*. The parallel conductive paths cause the resistivity to decrease slowly. It is worth noting that the above results were derived from an arbitrary microscopic unit area within the composite material, where the number of R_C_ is a small finite value *n*. Critically, R_C_ in different microscopic unit areas may form parallel connections in varying configurations, meaning the value of *j* can differ across microscopic unit areas. Consequently, the final result for the entire composite can be approximated as the sum of series and parallel resistances from numerous microscopic unit areas, each with different parallel groupings *j*, where *j* varies from 2 to *n* − *m*. When the CNT weight percentage exceeds 20 wt%, a large number of CNTs come into contact with each other, making parallel conductive paths gradually dominant, and the conductive network approaches saturation. At this time, the resistivity no longer changes significantly with CNT content but remains at a low value. Figure 4c shows the change in surface resistivity of composite films with 11.24 wt% CNTs during 20,000 bending cycles. It can be seen that the surface resistivity remains basically unchanged after multiple bending cycles, demonstrating excellent mechanical stability.

### 3.2. Piezoresistive Characteristics and Sensing Mechanisms of CP1–CP5

**Figure 5:**
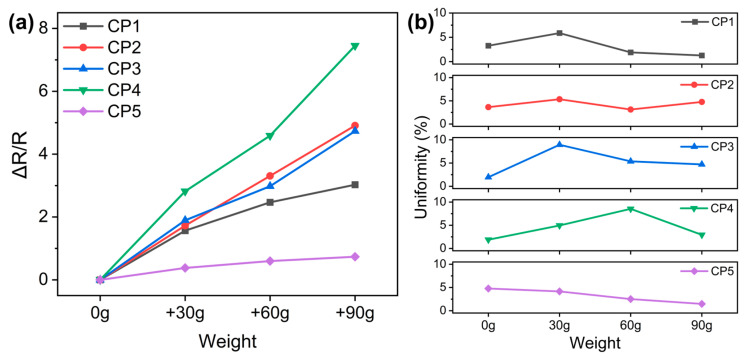
(**a**) ∆R/R–pressure curves of CP1–CP5; (**b**) the uniformity of steady resistance across each segment.

To select the most suitable CNT/PVA ratio for sensor fabrication, identical tests were performed on CP1–CP5 as follows: three 30 g weights were sequentially placed on the sensor while measuring its resistance changes, and step curves of resistance decrease were plotted for each weight addition. Based on the sensitivity (S) calculation, we obtain Formula (5):

(5) $$S=\frac{\delta \left[∆\frac{1}{R}/\frac{1}{R_{0}}\right]}{\delta P}=\frac{\delta [∆R/R]}{\delta P}$$

where $∆\frac{1}{R}$ equals $\frac{1}{R}$ minus $\frac{1}{R_{0}}$, R is the resistance with applied pressure (P), and R_0_ is the initial resistance without external pressure. $∆\frac{1}{R}/\frac{1}{R_{0}}$ represents the relative change rate of resistance reciprocal, which can be mathematically transformed into ∆R/R. $\frac{\delta \;[∆R/R]}{\delta P}$ represents the ratio of the relative change rate in the sensor’s output signal to the variation in applied pressure, which constitutes the physical meaning of sensitivity (S). The average value of each stabilized resistance data segment was used to calculate ∆R/R, which was subsequently plotted against weight to generate the ΔR/R-versus-weight curve, with results shown in Figure 5a. The sensitivity was ultimately determined as the slope of the curve. Based on the uniformity calculation, we obtain Formula (6):

(6) $$\mathrm{Uniformity}=\frac{\mathrm{Max}-\mathrm{Min}}{2\;\times \;\mathrm{Average}}\;\times \;100\%$$

The uniformity of steady resistance values for each segment is demonstrated in Figure 5b, which was employed to characterize the dispersion degree of multiple datasets acquired from each sensor under different constant loads, confirming the authenticity and reliability of the data. The results demonstrate that CP4 exhibits the largest curve slope, indicating the highest sensitivity. Furthermore, CP4 maintains a significant resistance change rate even in the high-pressure range (60–90 g), demonstrating excellent responsiveness across a wide pressure range. Figure 5b serves as a supplementary analysis to Figure 5a, which serves two critical purposes: (1) it demonstrates excellent signal stability with minimal resistance variation (low uniformity values) under constant loading; and (2) it confirms the representativeness and reliability of results obtained through averaging extensive datasets under identical experimental conditions.

**Figure 6:**
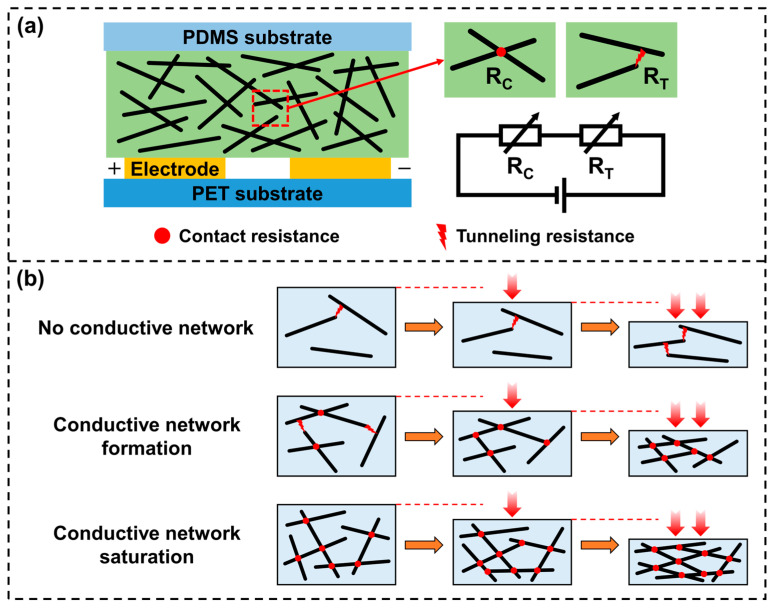
(**a**) Resistance model of the sensor; (**b**) working mechanisms of sensors with different CNT contents.

Figure 6 explains the sensing mechanism of the sensor and the intrinsic relationship between the above results and the CNT/PVA ratio. The variable resistance inside the sensor consists of two parts: the contact resistance R_C_ formed by the contact between CNTs in the sensing layer, and the tunneling resistance R_T_ generated by the tunneling effect when adjacent CNTs are very close to each other, as shown in Figure 6a. When the sensor is subjected to external force, the sensing layer is compressed and deformed, causing some CNTs inside to come into physical contact, thereby generating inter-tube contact resistance R_C_. For other CNTs that may still not be in contact, when the spacing is compressed small enough, tunneling current will form between CNTs due to the tunneling effect. This tunneling resistance R_T_ generated by the tunneling effect is positively correlated with the spacing between conductive particles and often decreases with increasing material compression.

Combining Figure 6a with the previously shown Figure 4b allows for further analysis of the mechanisms of different sensors, demonstrating their correlation with CNT content in the materials, which primarily manifests in how much the conductive paths in the composite change under external force, as illustrated in Figure 6b. For sensor CP5, the low CNT content results in large inter-particle distances, making it difficult for CNTs to come into contact even when the sensing layer undergoes significant deformation under high pressure. Conduction can only occur through the tunneling effect between a few closely spaced CNTs, leading to consistently high resistance with minimal variation. For sensor CP4, the CNT content falls within the range where conductive networks begin to form (the percolation zone). When subjected to pressure, the average distance between CNTs decreases, which leads to a rapid increase in physical contact points and the consequent formation of additional conductive pathways. As the material continues to compress under pressure, the sensing network evolves from initial formation to gradual saturation, resulting in substantial resistance changes and demonstrating significant piezoresistive characteristics. For CP1 and CP2, the sensing layers contain higher CNT contents where numerous contacts already exist without external force, forming nearly saturated conductive networks with low initial resistance. When external force is applied, the additional CNT contacts are negligible compared to the existing ones, and changes in the conductive network have minimal effect on resistance. The combination of low initial resistance due to high CNT content and small resistance variations makes these compositions unsuitable for sensor fabrication.

### 3.3. Characterization and Effect of Microstructures

**Figure 7:**
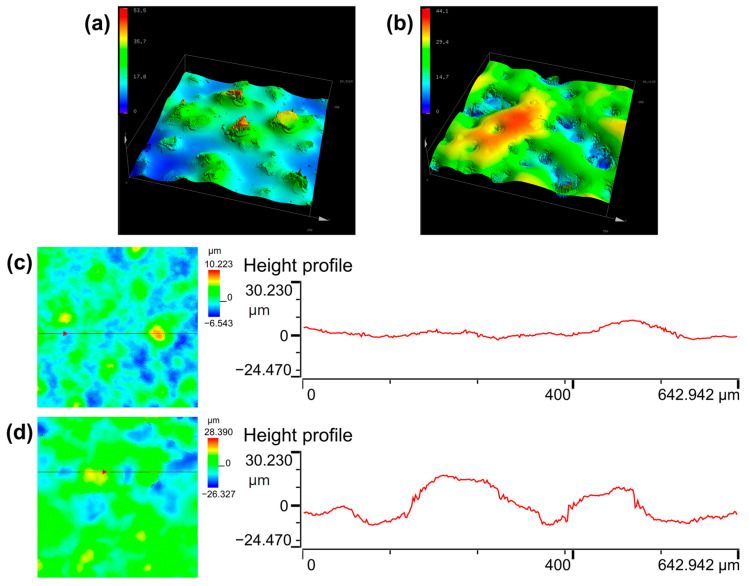
Laser confocal microscopy images: (**a**,**b**) three-dimensional morphological analysis of sandpaper and microstructured substrate; (**c**,**d**) height profiles of CP4 and M-CP4.

Although the CNT/PVA sensing layer in the percolation zone exhibits the best piezoresistive characteristics under external force, the deformation capability of planar structures (more specifically, non-microstructured sensor) is very limited due to the limitation imposed by the elastic resistance of the elastomer, which can only induce minimal increases in contact area and result in typically poor sensor performance [7,28]. To further enhance its performance, we used 800-grit sandpaper as a template to prepare PDMS substrates with microstructures through a single transfer process. The three-dimensional morphology analysis of the sandpaper is shown in Figure 7a, revealing surface protrusions of varying shapes, sizes, and heights. The same characterization of the microstructured PDMS substrate is shown in Figure 7b. After a single transfer, the protruding particles on the sandpaper transformed into continuous hill-like microstructures, with most hills measuring 30–60 μm in width and 15–30 μm in height.

Figure 3h shows the surface morphology of the M-CP4 sensing film observed under laser confocal microscopy. Comparing this figure with Figure 3e, it can be seen that M-CP4 has a rougher surface structure, exhibiting mountain-like morphological characteristics. Figure 7c and Figure 7d, respectively, display the height profiles of CP4 and M-CP4 films, showing that M-CP4 exhibits significantly greater surface undulation than CP4. This is partly due to the inherent unevenness of the microstructured substrate itself. In addition, during the film formation process, the hill-like microstructures may have caused more non-uniform distribution and deposition of solutes within small areas. It is worth noting that although CP4 was prepared on a flat substrate, its film surface is not completely smooth. On one hand, this is because the CNTs acting as conductive fillers are not fully encapsulated within the PVA matrix, but rather partially exposed on the surface to form conductive points; on the other hand, during the drop-coating and drying process, the difference in droplet evaporation rates induces surface-driven forces that lead to non-uniform deposition of solute particles [43,44]. Here, we can define the influence of surface undulation on performance by the ratio of height difference (Δh) of undulation to the film’s original thickness (ω, ω = 30 μm). When the Δh/ω value is less than 0.5, such undulation can be considered insufficient to affect performance. As can be seen from Figure 7c,d, the height difference in CP4 is only about 10 μm, with the Δh/ω value of CP4 obviously less than 0.5, confirming its negligible impact on performance. Whereas for M-CP4, the height difference reaches around 30 μm, with the Δh/ω value approximating 1, thereby significantly affecting performance. Therefore, when analyzing the sensor’s performance, CP4 can still be treated as a planar film compared to that of the hill-like microstructures in M-CP4.

**Figure 8:**
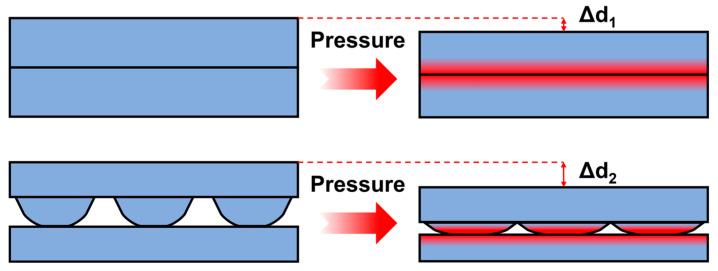
Deformation comparison between planar and hill-like microstructures under pressure.

Numerous studies have demonstrated that microstructures with different shape characteristics have varying effects on sensor performance [45,46]. Huang et al. [7] proposed that microstructures with a small shape factor (e.g., cones, spikes) can undergo significant deformation under minimal pressure, substantially improving sensitivity. In contrast, microstructures with a large shape factor (e.g., waves, ridges) can sustain continuous deformation across a wide pressure range, helping to expand the detection range. Theoretically, regardless of microstructure type, all can improve sensitivity to varying degrees because they exhibit stronger deformation capability than planar structures under given loads, thereby inducing more obvious changes in electrical parameters. For piezoresistive sensors, this typically manifests as (1) a significant increase in contact area leading to expanded conductive pathways; or (2) substantial deformation triggering the percolation effect or tunneling effect. The hill-like structures employed in this study clearly belong to the category of large shape factor microstructures, which provide the sensing layer with greater deformation space and enhanced compression capacity when subjected to force, facilitating stress dispersion and release. Consequently, they avoid saturation caused by localized stress concentration, thereby achieving a broader sensing range, as illustrated in Figure 8.

### 3.4. Sensing Performance of M-CP4

The sensor performance was tested using a digital multimeter and a universal testing machine. The sensitivity measuring procedure was conducted as follows:

(1) Pressure Application and Data Acquisition: A universal testing machine was first used to apply gradually increasing pressure to the sensor, while a digital multimeter monitored the corresponding resistance changes. At each pressure level, a segment of stable resistance data was recorded;
(2) Data Processing: The average value of each stable resistance segment was then calculated and defined as the resistance value corresponding to that pressure;
(3) Sensitivity Calculation: According to Formula (5) listed before, the relative change rate of resistance reciprocal (∆R/R) was computed for each pressure point. These values were plotted to generate a ΔR/R-versus-pressure (P) curve, and the sensitivity was ultimately determined as the slope of the linear fit to this curve.

**Figure 9:**
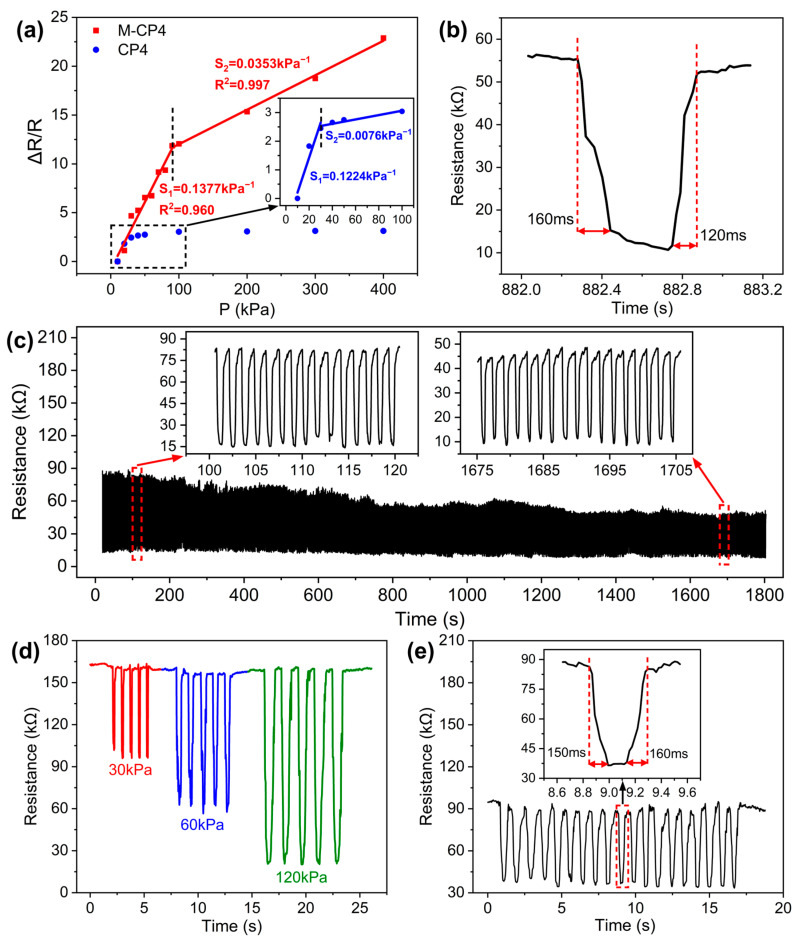
(**a**) Comparison of sensitivity and sensing range between M-CP4 and CP4; (**b**) response and recovery time of M-CP4 (The black line represents the output signal curve, while the red dotted lines and arrows indicate the response and recovery time ranges); (**c**) loading–unloading cyclic tests of M-CP4 under 0–90 kPa; (**d**) repeated responses of M-CP4 under different pressures; (**e**) loading–unloading cyclic tests of M-CP4 after 2000 folding cycles.

As shown in Figure 9a, M-CP4 demonstrates improved initial sensitivity compared to CP4 (increased from 0.1224 kPa^−1^ to 0.1377 kPa^−1^) and a three-times-higher threshold pressure (the pressure at which initial sensitivity begins to decline, increased from 30 kPa to 90 kPa). This enhancement stems from the hill-like microstructures maintaining superior deformation capacity under increasing pressure, whereas planar structures become nearly incompressible. Furthermore, M-CP4 retains relatively high sensitivity (0.0353 kPa^−1^) even beyond the threshold pressure, which enables an exceptionally wide linear working range up to 400 kPa suitable for diverse practical applications. Table 2 compares M-CP4 with recently reported CNT/polymer composite-based sensors, highlighting its superior sensitivity and ultra-wide sensing range achieved with simple materials, demonstrating outstanding overall performance.

Figure 9b shows that M-CP4 exhibits fast response time (160 ms) and recovery time (120 ms), meeting requirements for rapid response and recognition. Figure 9c presents the loading–unloading cyclic tests of M-CP4 under 0–90 kPa, where the sensor maintains high resistance variation after 1200 cycles, indicating excellent cycling stability and repeatability. Figure 9d displays the repeated responses of M-CP4 under different pressures, demonstrating its outstanding dynamic response and discrimination capability. The M-CP4 sensor was subjected to 2000 folding cycles under no-load conditions, followed by loading–unloading cyclic tests between 0 and 90 kPa. Figure 9e reveals that M-CP4 maintains excellent rapid repeatable response capability with nearly unchanged response and recovery time. This confirms that the conductive network within the sensitive layer remains undamaged, and the sensor possesses exceptional reliability and durability for long-term use in flexible electronic devices.

### 3.5. Practical Application of M-CP4 in Human Motion Detection

**Figure 10:**
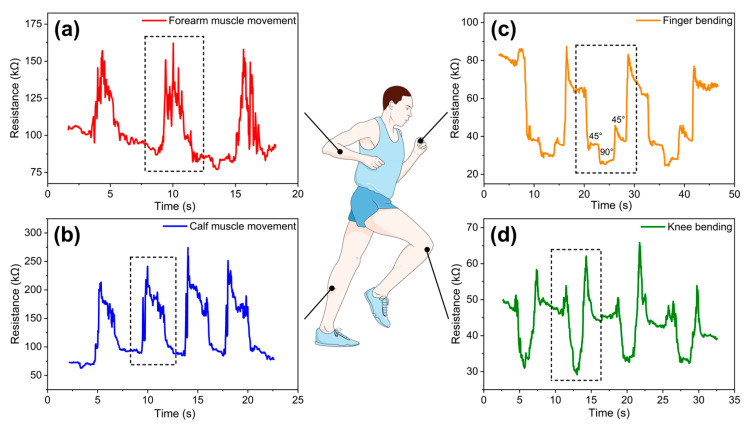
Practical application tests of M-CP4 (The black dotted boxes indicate the output signal variations corresponding to each repeated motion): (**a**,**b**) muscle activities of forearm and calf; (**c**,**d**) bending of finger and knee.

Given its excellent sensing performance, M-CP4 can accurately detect various human movements, as shown in Figure 10. The sensor was placed on the forearm and calf, respectively. When controlling muscle contraction and relaxation in corresponding areas, the skin applies varying pressure to the sensor, generating distinct physiological signals (Figure 10a,b). With the sensor attached to the index finger joint, the resistance value shows regular decreasing and increasing curves as the finger bends at different angles (Figure 10c). When fixed on the knee, the sensor’s resistance value also changes significantly during knee flexion and extension (Figure 10d). These test results demonstrate the sensor’s great application potential in healthcare monitoring, wearable devices, and related fields.

## 4. Conclusions

In summary, this study prepared a flexible piezoresistive sensor with hill-like microstructures and a wide linear detection range based on CNT/PVA composites. During the research process, we obtained the following key results:

(1) We systematically investigated the effect of CNT/PVA ratio on the sensor’s piezoresistive characteristics, finding that the sensing film exhibits optimal piezoresistive properties when the weight percentage of CNTs reaches 11.24 wt%;
(2) We proposed a piezoresistive model for the composite conductive material and explained the working mechanism related to CNT content, thereby further confirming that conductive networks in the percolation zone exhibit the best sensing performance;
(3) By further combining with PDMS substrates featuring hill-like microstructures, we achieved a dual improvement in both sensor sensitivity and detection range. The optimized sensor demonstrates a sensitivity of 0.1377 kPa^−1^ in the 0–90 kPa range, a wide linear working range exceeding 400 kPa, along with short response time (160 ms), good cycling stability (over 1200 pressure cycles), and flexibility stability (over 2000 folding cycles);
(4) The sensor can successfully detect various human activities, including joint bending (finger and knee flexion) and muscle movement (arm and leg muscle contractions).

This work provides a feasible solution for low-cost preparation of high-performance flexible electronic sensors, showing broad application prospects in wearable devices, smart healthcare monitoring, and human–machine interaction fields.

## Figures and Tables

**Table 1:** Sample number.

Sample Number	CP0	CP1	CP2	CP3	CP4	CP5	M-CP0	M-CP4
Substrate	Flat substrate						Microstructured substrate	
CNT dispersions/PVA solution volume ratio	1:0	2:1	3:2	1:1	2:3	1:2	1:0	2:3
CNT weight percentage (wt%)	100.00	27.54	22.18	15.97	11.24	8.68	100.00	11.24

**Table 2:** Performance comparison between M-CP4 and other reported sensors based on CNT/polymer composites.

Sensing Materials	Maximum Sensitivity	Working Range	Refs.
CNT-PDMS sponge	0.033 kPa^−1^	55 kPa	[47]
CNT/PDMS	0.59 kPa^−1^	260 kPa	[48]
nano-ZnO/CNT/PDMS	0.18 kPa^−1^	80 kPa	[40]
CNT/CB/CIP/silicone	0.136 kPa^−1^	220 kPa	[49]
CNT/CB/TPU@PU	0.1 kPa^−1^	23.3 kPa	[50]
CNT/PVA	0.1377 kPa^−1^	400 kPa	This work

## Data Availability

The original contributions presented in this study are included in the article. Further inquiries can be directed to the corresponding author.
